# Surgical resection of nodular ground-glass opacities without percutaneous needle aspiration or biopsy

**DOI:** 10.1186/1471-2407-14-838

**Published:** 2014-11-18

**Authors:** Jaeyoung Cho, Sung-Jun Ko, Se Joong Kim, Yeon Joo Lee, Jong Sun Park, Young-Jae Cho, Ho Il Yoon, Sukki Cho, Kwhanmien Kim, Sanghoon Jheon, Jae Ho Lee, Choon-Taek Lee

**Affiliations:** Department of Internal Medicine, Seoul National University College of Medicine, Seoul, Korea; Division of Pulmonology and Critical Care Medicine, Department of Internal Medicine, Seoul National University Bundang Hospital, Seongnam, Korea; Department of Thoracic and Cardiovascular Surgery, Seoul National University College of Medicine, Seoul, Korea; Department of Thoracic and Cardiovascular Surgery, Seoul National University Bundang Hospital, Seongnam, Korea

**Keywords:** Nodular ground-glass opacity, Lung cancer, Computed tomography, Surgery, Percutaneous needle aspiration or biopsy

## Abstract

**Background:**

Percutaneous needle aspiration or biopsy (PCNA or PCNB) is an established diagnostic technique that has a high diagnostic yield. However, its role in the diagnosis of nodular ground-glass opacities (nGGOs) is controversial, and the necessity of preoperative histologic confirmation by PCNA or PCNB in nGGOs has not been well addressed.

**Methods:**

We here evaluated the rates of malignancy and surgery-related complications, and the cost benefits of resecting nGGOs without prior tissue diagnosis when those nGGOs were highly suspected for malignancy based on their size, radiologic characteristics, and clinical courses. Patients who underwent surgical resection of nGGOs without preoperative tissue diagnosis from January 2009 to October 2013 were retrospectively analyzed.

**Results:**

Among 356 nGGOs of 324 patients, 330 (92.7%) nGGOs were resected without prior histologic confirmation. The rate of malignancy was 95.2% (314/330). In the multivariate analysis, larger size was found to be an independent predictor of malignancy (odds ratio, 1.086; 95% confidence interval, 1.001-1.178, p =0.047). A total of 324 (98.2%) nGGOs were resected by video-assisted thoracoscopic surgery (VATS), and the rate of surgery-related complications was 6.7% (22/330). All 16 nGGOs diagnosed as benign nodules were resected by VATS, and only one patient experienced postoperative complications (prolonged air leak). Direct surgical resection without tissue diagnosis significantly reduced the total costs, hospital stay, and waiting time to surgery.

**Conclusions:**

With careful selection of nGGOs that are highly suspicious for malignancy, surgical resection of nGGOs without tissue diagnosis is recommended as it reduces costs and hospital stay.

## Background

With the recent technological and diagnostic advances, and with the widespread use of computed tomography (CT), nodular ground-glass opacities (nGGOs) are being increasingly detected. This increased detection has challenged the diagnosis and management of nGGOs, which have been addressed extensively in the last ten years. However, most approaches are currently not closely grounded in solid evidence [1]. The most recent National Comprehensive Cancer Network (NCCN) guidelines, version 2.2014 for lung cancer screening recommend nonsurgical biopsy or surgical excision of part-solid nodules measuring more than 8 mm that are suspicious for lung cancer upon fluorodeoxyglucose-positron emission tomography (FDG-PET)/CT [2]. For pure GGOs measuring more than 10 mm with stable features upon low-dose CT (LDCT) follow-up, the NCCN guidelines recommend follow-up with LDCT for 6–12 months, nonsurgical biopsy, or surgical excision. On the other hand, in patients with part-solid nodules measuring more than 8 mm, the American College of Chest Physicians (ACCP) guidelines suggest repeat chest CT at 3 months, followed by further evaluation with FDG-PET/CT, nonsurgical biopsy, and/or surgical resection [3]. Especially, in part-solid nodules measuring more than 15 mm, they recommend prompt further evaluation with FDG-PET/CT, nonsurgical biopsy, and/or surgical resection. However, there are currently no clear criteria for deciding nonsurgical biopsy or surgical resection.

Bronchoscopic examination including biopsy is rarely helpful in the diagnosis of nGGOs, as these are usually located peripherally. On the other hand, percutaneous needle aspiration or biopsy (PCNA or PCNB) is an established diagnostic technique that has a high diagnostic yield [4, 5]; however, PCNA or PCNB has a lower sensitivity in smaller nodules [6, 7], and its role in the diagnosis of nGGOs is controversial, with no consensus existing regarding the optimal size threshold or technique [8]. Although recent studies have reported that PCNB provided a high diagnostic accuracy of up to 95% for nGGOs [9, 10], those rates may not be reproducible when strict standards are applied to the biopsy specimens under the new classification of the International Association for the Study of Lung Cancer/American Thoracic Society/European Respiratory Society (IASLC/ATS/ERS), which introduced the concept of adenocarcinoma in situ (AIS) and minimally invasive adenocarcinoma (MIA) [11]. Small biopsy may not be suitable for determining tissue invasiveness.

In addition to pneumothorax or hemoptysis, the risk of malignant cell spread through the tract upon PCNA or PCNB has been addressed [12–15]. Furthermore, the relatively high radiation exposure to the operators is a concern, as percutaneous biopsy of nGGOs is associated with a longer procedure time, owing to the smaller sizes and less solid components of these tumors.

Thin-section CT findings correlate closely with the pathologic diagnosis of nGGOs [16], and the attenuation, marginal characteristics, size, and development of a solid component are potentially helpful to predict malignancy [1, 17, 18]. Therefore, surgical resection of nGGOs without preoperative tissue diagnosis could be a reasonable strategy. However, the necessity of preoperative histologic confirmation by PCNA or PCNB in nGGOs has not been well evaluated.

**Figure 1:**
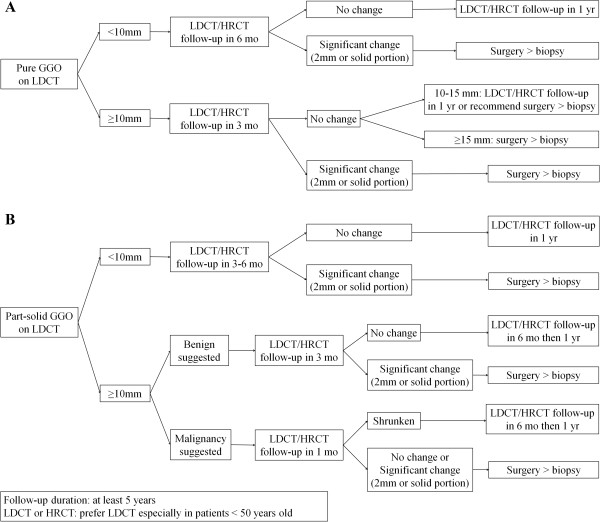
**Seoul National University Bundang Hospital guidelines for pure GGO (A) and part-solid GGO (B).** GGO, ground-glass opacity; LDCT, low-dose computed tomography; HRCT, high resolution computed tomography.

Seoul National University Bundang Hospital (SNUBH) has used its own protocol for the management of nGGOs (Figure 1) that is basically similar to the NCCN guideline [2]. Briefly, we suggest direct surgical resection of nGGOs highly suspicious for malignancy, rather than nonsurgical biopsy. In this study, we evaluated the rate of malignancy, complications related to surgery, and the cost benefits of resecting nGGOs without prior tissue diagnosis when those nGGOs were highly suspected for malignancy based on their size, radiologic characteristics, and clinical courses.

## Methods

### Study population and study design

We retrospectively reviewed the medical records of all patients who underwent surgical resection of nGGOs between January 2009 and October 2013 at SNUBH in Seoul, Korea. Patients who underwent preoperative tissue diagnosis by PCNA or PCNB were excluded. The primary outcomes were the rate of malignancy of nGGOs among patients who underwent surgical resection without prior tissue diagnosis, and the clinical and radiological predictors of malignancy. The secondary outcomes were complications related to surgery and differences in the total costs, days of hospitalization, and the time interval before surgery between patients with or without nonsurgical histologic diagnosis. This study was approved by the institutional review board of SNUBH (L-2014-274). The requirement for informed consent was waived.

### Radiologic evaluation

CT scans were obtained using various instruments, including the Brilliance-64, MX-8000 IDT, and iCT 256 (Philips Medical Systems, Cleveland, OH, USA). Imaging was obtained using a lung window setting with a level of -600 Hounsfield units (HU) and a width of 1500 HU, and a mediastinal window setting with a level of 30 HU and a width of 400 HU. Scanning was performed from the thoracic inlet to the upper portion of the kidneys. When there were multiple nGGOs in a patient, only nGGOs with permanent pathologic confirmation were selected based on the surgical records. The nGGO lesions containing patchy opacities that totally obscured the lung parenchyma were classified as part-solid GGOs, whereas if no part of the encircled lung parenchyma was completely obscured, they were classified as pure GGOs [19]. In each nGGO, the presence of a solid component, an air-bronchogram, bubble lucency, pleural or fissure retraction, and margin irregularity were evaluated. The maximal diameters and tumor disappearance rate were also measured [20]. In 191 nGGOs of 173 patients who underwent FDG-PET/CT, data on the maximal standardized uptake values (SUVs) were also collected.

Serial CT scans performed at least every 4 weeks were available for 244 nGGOs in 223 patients over a median follow-up duration of 9.1 months (range, 7.3-123.9 months). The interval change was investigated in these nGGOs, and progression of nGGO was defined as (1) ≥2 mm increase in the GGO size, (2) ≥2 mm increase in the solid component, or (3) emerging new solid component of any size [21, 22].

### Total costs, days of hospitalization, and waiting time

Total costs were calculated as the sum of the costs for both the diagnosis and treatment of nGGOs during hospitalization. We excluded all charges incurred in the outpatient clinic. Costs were converted to US dollars according to the current average exchange rate (1 US dollar =1025 won). Days of hospitalization were defined as the total length of hospital stay for both surgical resection and work-up. Waiting time (the time interval before surgery) was defined as the interval between the first hospital day of admission for work-up and the day of operation, even if a patient was discharged and readmitted for surgery [23].

### Statistical analysis

Continuous data are presented as mean ± standard deviation (SD), whereas categorical data are presented as numbers and percentages. The relationships between the clinical and radiological characteristics and the final pathologic diagnosis were evaluated using the independent-sample t-test for continuous variables and the χ2 test or Fisher’s exact test for categorical variables. Univariate and multivariate analyses were performed and the results were described with odds ratio (OR) and 95% confidence interval (CI). P values less than 0.05 were considered to have statistical significance. All statistical analyses were performed using SPSS for Windows, version 19.0 (SPSS Inc., Chicago, IL, USA).

## Results

### Patient characteristics

**Figure 2:**
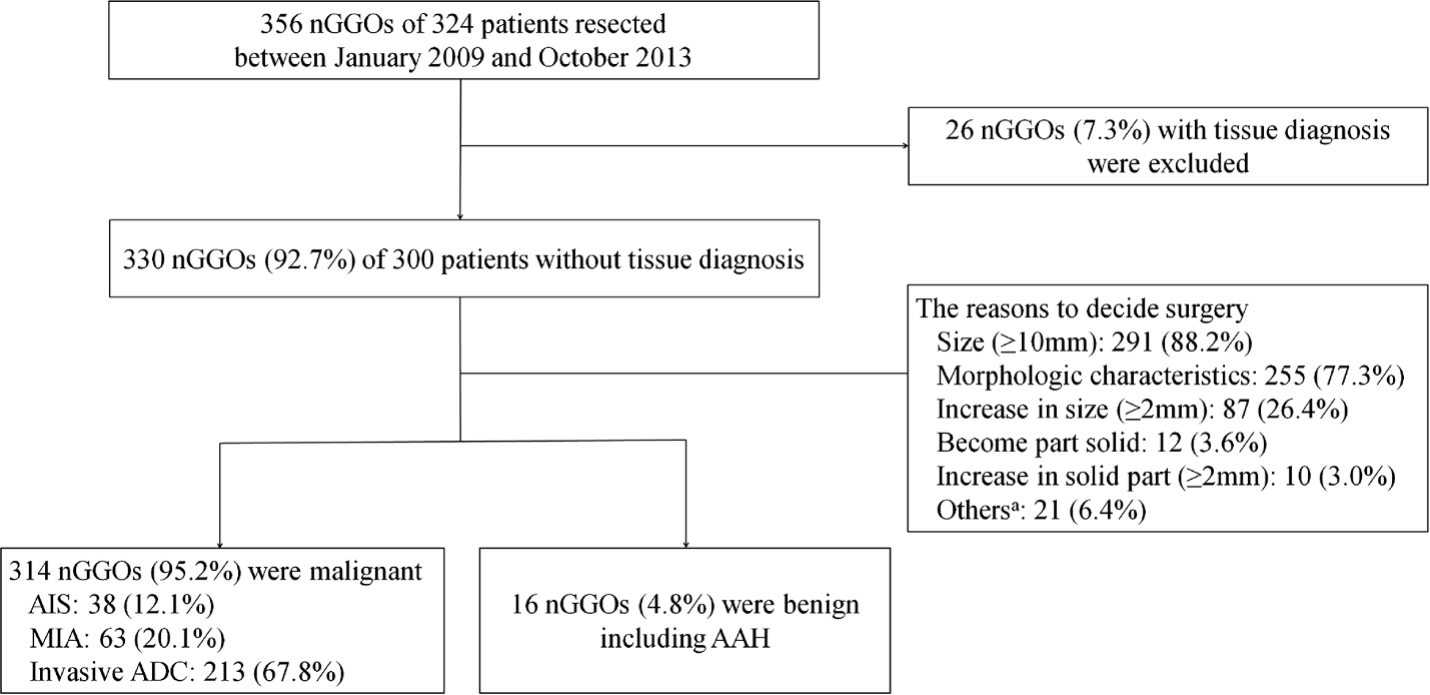
**Flow chart.** Of the 330 resected nGGOs, 314 were diagnosed as lung adenocarcinoma, and the rate of malignancy was 95.2%. ^a^Among 21 nGGOs, six nGGOs were co-resected with indicated GGO nodules and one nGGO was resected because metastasis of underlying thyroid cancer was strongly suspected. The decision to resect the other 14 GGOs was mainly influenced by the patients’ will. nGGOs, nodular ground-glass opacities; AIS, adenocarcinoma in situ; MIA, minimally invasive adenocarcinoma; ADC, adenocarcinoma; AAH, atypical adenomatous hyperplasia.

Of 356 nGGOs in 324 patients who underwent surgical resection for nGGOs from January 2009 and October 2013, 330 nGGOs (92.7%) in 300 patients were resected without preoperative tissue diagnosis (Figure 2). The main indications for direct surgical resection were a tumor size more than 10 mm (n =291, 88.2%), morphologic characteristics (an air-bronchogram, bubble lucency, pleural or fissure retraction, or irregular margin) on CT scans (n =255, 77.3%), and an increase ≥2 mm in the whole GGO size (n =87, 26.4%). All other reasons that are not indicated by our protocol are listed in Figure 2. Among 21 nGGOs, six nGGOs were co-resected with indicated GGO nodules and one nGGO was resected because metastasis of underlying thyroid cancer was strongly suspected. The decisions to resect other nGGOs that did not meet the SNUBH protocol were mainly influenced by the patients’ will.

Of the 330 resected nGGOs, 314 were diagnosed as lung adenocarcinoma, and the rate of malignancy was 95.2%. A total of 242/255 (94.9%) part-solid GGOs, and 72/75 (96.0%) pure GGOs were malignant lesions. Only 16 nGGOs (4.8%) were proven to be benign lesions, including atypical adenomatous hyperplasia (AAH). Regarding the demographic characteristics, there was no significant differences in either age (p =0.127) or sex (p =0.461) between patients with malignant and benign lesions (Table 1). Moreover, smoking history (p =0.555) did also not differ between these groups.

**Table 1:** **Baseline and radiologic characteristics of the study patients (n =330)**

	Benign	Malignant	***p***-value
	(n =16)	(n =314)	
**Baseline characteristics**			
Age (years)^a^	58.1 ± 7.3	62.1 ± 10.4	0.127
Male sex, no. (%)	9 (56.3)	147 (46.8)	0.461
Smoking, no. (%)			0.555
Never-smoker	8 (50.0)	196 (62.4)	
Ex-smoker	6 (37.5)	95 (30.3)	
Current-smoker	2 (12.5)	23 (7.3)	
Smoking (PY)	12.2 ± 17.3	10.0 ± 16.5	0.605
**Radiologic characteristics**			
Size (mm)^a^	15.1 ± 9.3	20.3 ± 11.0	0.063
GGO pattern, no. (%)			1.0
Pure	3 (18.8)	72 (22.9)	
Part-solid	13 (81.3)	242 (77.1)	
TDR (%)^a^	86.6 ± 17.5	83.3 ± 20.7	0.536
Air bronchogram, no. (%)	7 (43.8)	175 (55.7)	0.347
Bubble lucency, no. (%)	2 (12.5)	46 (14.6)	1.0
Pleural or fissure retraction, no. (%)	4 (25.0)	143 (45.5)	0.107
Irregular margin, no. (%)	6 (37.5)	148 (47.1)	0.451
Maximal SUV on FDG-PET/CT^a,b^	0.39 ± 0.67	1.19 ± 1.28	0.101
Progression, no. (%)^c^			1.0
Progression	4/12 (33.3)^d^	88/232 (37.9)	
Without progression	8/12 (66.7)	144/232 (62.1)^e^	

^a^Expressed as mean values ± standard deviations.

^b^Among the 330 nGGOs, 191 underwent FDG-PET/CT.

^c^Serial CT scans at least 4 weeks interval were available for 244 nGGOs over the median follow-up duration of 9.1 months (range, 7.3 - 123.9 months).

^d^Three GGOs had increased in whole GGO size and one pure GGO had become a part solid nodule. The two of three GGOs were subpleural fibrosis and the other one was atypical adenomatous hyperplasia on the final pathology. The one GGO becoming a part solid nodule was the anthracofibrotic nodule.

^e^One GGO had decreased in size, which was adenocarcinoma, acinar predominant.

PY, pack-years; GGO, ground-glass opacity; TDR, tumor disappearance rate; SUV, maximal standardized uptake value; FDG-PET/CT, fluorodeoxyglucose-positron emission tomography/computed tomography.

### Radiologic characteristics

The mean values of the maximal diameter ± SD of the nGGOs were 15.1 ± 9.3 mm and 20.3 ± 11.0 mm for benign and malignant lesions, respectively (p =0.063) (Table 1). The morphologic characteristics of the nGGOs in terms of the presence of a solid component, an air-bronchogram, bubble lucency, pleural or fissure retraction, and margin irregularity did not significantly differ between the groups. Moreover, for the 191 nGGOs with FDG-PET/CT findings, the maximal SUV was not significantly different between benign and malignant lesions.

Of 244 nGGOs followed-up over 9.1 months (range, 7.3-123.9 months), 92 (37.7%) showed tumor progression. However, progression of nGGOs was not associated with the risk of malignancy. Of 12 benign nGGOs, four (33.3%) showed progression. Three nGGOs displayed increases in the whole GGO size, and the one pure GGO had become a part-solid GGO. Two of the three nGGOs displaying increases in the whole GGO size were subpleural fibrosis, whereas the other one was diagnosed as AAH on final pathology. The one nGGO becoming a part-solid nodule turned out to be an anthracofibrotic nodule. On the other hand, one nGGO had decreased in size, which was an adenocarcinoma, acinar predominant.

In the multivariate analysis, larger size was the only identified independent factor that was predictive of malignancy (OR, 1.086; 95% CI, 1.001-1.178; p =0.047). Especially, nGGOs more than 15 mm in size were significantly associated with a higher risk of malignancy compared to nGGOs less than 10 mm in the multivariate analysis (OR, 8.323; 95% CI, 1.968 – 35.196; p =0.004) (Table 2).

**Table 2:** **Multivariate analysis for the risk of malignancy according to ground glass opacity size**

Size	Total	Benign	Malignant	p-value	OR	95% CI
	(n =330)	(n =16)	(n =314)			
< 10 mm	39	5 (31.3)	34 (10.8)	0.015		
10 mm ≤ <15 mm	76	5 (31.3)	71 (22.6)	0.146	2.777	0.701 – 10.994
15 mm ≤	215	6 (37.5)	209 (66.6)	0.004	8.323	1.968 – 35.196

OR, odds ratio; CI, confidence interval.

### Surgical procedure and complications related to surgery

Limited surgical resection was conducted in 170/330 (51.5%) nGGOs. 324 nGGOs (98.2%) were resected by video-assisted thoracoscopic surgery (VATS), and conversion of VATS to open thoracotomy occurred in only 4 cases (1.2%). Complications related to surgery occurred in 6.7% (22/330) of cases. The most common complications were prolonged air leak for more than 7 days (n =15; 68.2%), pleural effusion (n =4; 18.2%), pneumothorax (n =2; 9.1%), and chylothorax (n =1; 4.5%). All 16 nGGOs diagnosed as benign lesions were resected by VATS, and only one of these patients experienced postoperative complications (prolonged air leak).

### Pathologic diagnosis

Table 3 shows the pathologic diagnoses of the patients. Of the 314 malignant nGGOs, 38 (12.1%) were diagnosed as AIS and 63 (20.1%) as MIA and 213 (67.8%) were diagnosed as invasive adenocarcinomas. Of 213 (67.8%) invasive adenocarcinomas, 115 (36.6%) were acinar predominant, 52 (16.6%) were papillary predominant, and 33 (10.5%) were lepidic predominant. The most common pathologic findings in the 16 benign nGGOs were focal interstitial fibrosis (n =5; 31.3%), AAH (n =4; 25.0%), and subpleural fibrosis (n =3; 18.8%).

**Table 3:** **Pathologic diagnoses**

**Malignant (n =314)**	**No.**	**%**
Adenocarcinoma in situ	38	12.1
Minimally invasive adenocarcinoma	63	20.1
Invasive adenocarcinoma		
Lepidic predominant	33	10.5
Acinar predominant	115	36.6
Papillary predominant	52	16.6
Micropapillary predominant	1	0.32
Solid predominant	2	0.64
Variants		
Mucinous adenocarcinoma	9	2.9
Enteric	1	0.32
**Benign (n =16)**	**No.**	**%**
Focal interstitial fibrosis	5	31.3
Atypical adenomatous hyperplasia	4	25.0
Subpleural fibrosis	3	18.8
Respiratory bronchiolitis with fibrosis and lymphocytic infiltration	1	6.3
Heavy lymphoplasma cell infiltration	1	6.3
Pulmonary lymphomatoid granulomatosis	1	6.3
Anthracofibrotic nodule with reactive pneumocytes	1	6.3

### Advantages on costs, hospital stay, and waiting time

Next, we compared the total costs, days of hospitalization, and the waiting times between patients who underwent surgical resection of nGGOs with or without preoperative tissue diagnosis. Surgical resection without histologic confirmation was found to be associated with significant decreases in the total costs, hospital stay, and waiting times (Table 4). Approximately 2,546 US dollars were saved by performing surgical resection without tissue diagnosis as compared to with tissue diagnosis (9,271 vs. 11,817 US dollars, p =0.004). Moreover, the mean hospital stay was 3.0 days shorter (6.8 vs. 9.8 days, p =0.015) and the mean waiting time was 6.3 days shorter (2.5 vs. 8.8 days, p =0.001) in patients who underwent surgical resection without tissue diagnosis.

**Table 4:** **Hospital stays, waiting times, and costs for patients undergoing surgical resection with and without tissue diagnosis**

	Without tissue diagnosis	With tissue diagnosis	p-value
	(n =305) ^a^	(n =26)	
Days of hospitalization (days)	6.8 ± 6.1	9.8 ± 4.3	0.015
The time interval before surgery (days)	2.5 ± 2.9	8.8 ± 8.5	0.001
Total costs (US dollars)	9271 ± 4430	11817 ± 2479	0.004

Unless otherwise specified, data are expressed as mean values ± standard deviations.

^a^Data were evaluated for 305 operations in 300 patients, which included 330 nGGOs. Five patients had surgical resection twice for different nGGOs.

## Discussion

The aim of this retrospective study was to investigate the necessity of preoperative biopsy for GGO nodules which were suspicious for malignancy. We evaluated the rate of malignancy, complications related to surgery, and the cost benefits of surgical resection of nGGOs without preoperative tissue diagnosis when those nGGOs were highly suspicious for malignancy in terms of size, radiologic characteristics, and clinical courses. Lack of adequate control group, such as randomly assigned GGO nodules with preoperative biopsy was the main limitation of this study. However, our study yielded four main findings: (1) the rate of malignancy was high (95.2%) in nGGOs highly suspected for malignancy based on clinical and radiologic characteristics; (2) only tumor size was a significant independent predictor of malignancy in the multivariate analysis; (3) the rate of complications related to surgery was low (6.7%), with no mortality and minimal morbidity; and (4) direct surgical resection without tissue diagnosis significantly reduced the total costs, days of hospitalization, and waiting time to surgery.

The role of PCNA or PCNB in the diagnosis of nGGOs remains limited [8]. Hur et al. [24] reported the sensitivity of CT fluoroscopy-guided needle biopsy as 67% for diagnosing malignancy in 28 patients with nGGO lesions. In another study of 40 individuals with nGGOs, the diagnostic yield of percutaneous CT-guided core biopsy was 84% (16/19), whereas it was non-diagnostic in three patients (16%) [25]. Two of these underwent surgical resection and were diagnosed as lung adenocarcinoma. In our study, 26 patients underwent PCNA or PCNB and the diagnostic accuracy was found to be 65.4% (17/26) under the IASLC/ATS/ERS classification of lung adenocarcinoma. All nGGOs that were resected after PCNA or PCNB were in fact malignant. Four nGGOs diagnosed as benign lesions and another four nGGOs with non-diagnostic results on PCNA or PCNB underwent surgical resection based on strong clinical suspicion for lung cancer, and were demonstrated to be invasive adenocarcinomas.

Recently, several studies have shown that the diagnostic accuracy of PCNB was greater than 90% for nGGOs [9, 10]; however, these results may be dependent on the experience and skills of the operators, and may hence not be fully reproducible. In this context, the third edition of the ACCP guidelines on the diagnosis and management of lung cancer stated that nonsurgical biopsy should not be used to exclude malignancy considering its unsatisfactory sensitivity and limited negative predictive value [3].

VATS lobectomy for patients with early-stage lung cancer is a standard surgical treatment, and is associated with lower morbidity and improved survival rates compared with open thoracotomy [26]. Recently, several studies have suggested that thoracoscopic limited resection is a valid surgical technique for nGGOs selected by thin-section CT scans [27]. With the widespread use of VATS, it is possible to diagnose and treat nGGOs simultaneously.

Recommendations for the management of nGGOs have been used by SNUBH (Figure 1). These recommendations state that, regardless of the presence of a solid component, surgical resection should be considered if there is an increase in size ≥2 mm or development of a solid component in a pure GGO. In pure GGOs ≥10 mm, we suggest repeat chest CT at 3 months. In GGOs without significant changes in the initial 3 months of follow-up, we recommend surgical excision for nGGOs ≥15 mm, whereas we recommend chest CT follow-up for one year or surgical excision for nGGOs measuring 10–15 mm in size. In part-solid GGOs ≥10 mm with clinical suspicion of malignancy, we recommend surgical resection even if these do not show significant changes at the initial one-month follow-up.

Heo et al. [23] evaluated 113 patients who underwent surgical resection in SNUBH from January 2008 to May 2009 without prior tissue diagnosis for highly suspicious pulmonary nodules, including solid and GGO lesions. In their retrospective study, 45/50 (90%) patients with nGGOs had malignancy; and they reported that presence of a solid component, bubble lucency, irregular margin, and larger size correlated with malignancy. Although many other studies have also reported that larger size, irregular border, partly solid attenuation, internal air bronchograms, and central bubbly lucency were associated with higher rates of malignancy [19, 28, 29], some studies have reported conflicting results. In a study of 53 pure nGGOs in 49 patients, no significant differences in the morphologic characteristics and size were observed between malignant and benign nodules [30], although it is possible that this study was underpowered to detect differences [3]. However, in our study including a larger number of nGGOs, the maximal diameter was the only predictive factor of malignancy, and there were no significant differences in the morphologic features on CT between malignant and benign lesions.

We speculate that the failure to detect morphologic factors to distinguish benign from malignant nGGOs was mainly influenced by selection bias. Not all patients with an nGGO lesion underwent surgical resection. Only patients who were highly suspected to have malignancy based on the tumor size, radiologic characteristics, and clinical courses underwent surgical resection, although 14 of the resected nGGOs did not meet the SNUBH protocol in this study. This may explain the higher malignancy rate (95.2%) and lower proportion of AAH (4/330, 1.2%) in the present study compared to previous studies, which have reported malignancy rates between 58.7-75.0% [19, 30] and proportions of AAH between 5.7%-20.9% [30, 31].

## Conclusions

In conclusion, upon careful selection of nGGOs that are highly suspicious for malignancy, surgical resection of nGGOs without tissue diagnosis is recommended as it reduces costs and the length of hospital stays.

## Abbreviations

CT: Computed Tomography
nGGO: nodular Ground-Glass Opacity
NCCN: National Comprehensive Cancer Network
FDG-PET: Fluorodeoxyglucose-Positron Emission Tomography
LDCT: Low-Dose Computed Tomography
ACCP: American College of Chest Physicians
PCNA or PCNB: Percutaneous Needle Aspiration or Biopsy
IASLC: International Association for the Study of Lung Cancer
ATS: American Thoracic Society
ERS: European Respiratory Society
AIS: Adenocarcinoma in Situ
MIA: Minimally Invasive Adenocarcinoma
SNUBH: Seoul National University Bundang Hospital
HU: Hounsfield Unit
SUV: Standardized Uptake Value
SD: Standard Deviation
OR: Odds Ratio
CI: Confidence Interval
AAH: Atypical Adenomatous Hyperplasia
VATS: Video-Assisted Thoracoscopic Surgery.

## References

[1] Detterbeck FC, Lewis SZ, Diekemper R, Addrizzo-Harris D, Alberts WM (2013). Executive Summary: Diagnosis and management of lung cancer, 3rd ed: American College of Chest Physicians evidence-based clinical practice guidelines. Chest.

[2] **Lung cancer screening** [http://www.nccn.org]

[3] Gould MK, Donington J, Lynch WR, Mazzone PJ, Midthun DE, Naidich DP, Wiener RS (2013). Evaluation of individuals with pulmonary nodules: when is it lung cancer? Diagnosis and management of lung cancer, 3rd ed: American College of Chest Physicians evidence-based clinical practice guidelines. Chest.

[4] Hiraki T, Mimura H, Gobara H, Iguchi T, Fujiwara H, Sakurai J, Matsui Y, Inoue D, Toyooka S, Sano Y, Kanazawa S (2009). CT fluoroscopy-guided biopsy of 1,000 pulmonary lesions performed with 20-gauge coaxial cutting needles: diagnostic yield and risk factors for diagnostic failure. Chest.

[5] Montaudon M, Latrabe V, Pariente A, Corneloup O, Begueret H, Laurent F (2004). Factors influencing accuracy of CT-guided percutaneous biopsies of pulmonary lesions. Eur Radiol.

[6] Tsukada H, Satou T, Iwashima A, Souma T (2000). Diagnostic accuracy of CT-guided automated needle biopsy of lung nodules. AJR Am J Roentgenol.

[7] vanSonnenberg E, Casola G, Ho M, Neff CC, Varney RR, Wittich GR, Christensen R, Friedman PJ (1988). Difficult thoracic lesions: CT-guided biopsy experience in 150 cases. Radiology.

[8] Lorenz JM (2012). Updates in percutaneous lung biopsy: new indications, techniques and controversies. Semin Intervent Radiol.

[9] Inoue D, Gobara H, Hiraki T, Mimura H, Kato K, Shibamoto K, Iishi T, Matsui Y, Toyooka S, Kanazawa S (2012). CT fluoroscopy-guided cutting needle biopsy of focal pure ground-glass opacity lung lesions: diagnostic yield in 83 lesions. Eur J Radiol.

[10] Yamauchi Y, Izumi Y, Nakatsuka S, Inoue M, Hayashi Y, Mukai M, Nomori H (2011). Diagnostic performance of percutaneous core needle lung biopsy under multi-CT fluoroscopic guidance for ground-glass opacity pulmonary lesions. Eur J Radiol.

[11] Travis WD, Brambilla E, Noguchi M, Nicholson AG, Geisinger KR, Yatabe Y, Beer DG, Powell CA, Riely GJ, Van Schil PE, Garg K, Austin JH, Asamura H, Rusch VW, Hirsch FR, Scagliotti G, Mitsudomi T, Huber RM, Ishikawa Y, Jett J, Sanchez-Cespedes M, Sculier JP, Takahashi T, Tsuboi M, Vansteenkiste J, Wistuba I, Yang PC, Aberle D, Brambilla C, Flieder D (2011). International association for the study of lung cancer/american thoracic society/european respiratory society international multidisciplinary classification of lung adenocarcinoma. J Thorac Oncol.

[12] Voravud N, Shin DM, Dekmezian RH, Dimery I, Lee JS, Hong WK (1992). Implantation metastasis of carcinoma after percutaneous fine-needle aspiration biopsy. Chest.

[13] Yoshikawa T, Yoshida J, Nishimura M, Yokose T, Nishiwaki Y, Nagai K (2000). Lung cancer implantation in the chest wall following percutaneous fine needle aspiration biopsy. Jpn J Clin Oncol.

[14] Sawabata N, Ohta M, Maeda H (2000). Fine-needle aspiration cytologic technique for lung cancer has a high potential of malignant cell spread through the tract. Chest.

[15] Matsuguma H, Nakahara R, Kondo T, Kamiyama Y, Mori K, Yokoi K (2005). Risk of pleural recurrence after needle biopsy in patients with resected early stage lung cancer. Ann Thorac Surg.

[16] Kobayashi Y, Mitsudomi T (2013). Management of ground-glass opacities: should all pulmonary lesions with ground-glass opacity be surgically resected?. Transl Lung Cancer Res.

[17] Gandara DR, Aberle D, Lau D, Jett J, Akhurst T, Heelan R, Mulshine J, Berg C, Patz EF (2006). Radiographic imaging of bronchioloalveolar carcinoma: screening, patterns of presentation and response assessment. J Thorac Oncol.

[18] Park CM, Goo JM, Lee HJ, Lee CH, Chun EJ, Im JG (2007). Nodular ground-glass opacity at thin-section CT: histologic correlation and evaluation of change at follow-up. Radiographics.

[19] Lee HJ, Goo JM, Lee CH, Park CM, Kim KG, Park EA, Lee HY (2009). Predictive CT findings of malignancy in ground-glass nodules on thin-section chest CT: the effects on radiologist performance. Eur Radiol.

[20] Ko SJ, Lee YJ, Park JS, Cho YJ, Yoon HI, Chung JH, Kim TJ, Lee KW, Kim K, Jheon S, Kim H, Lee JH, Lee CT (2014). Epidermal growth factor receptor mutations and anaplastic lymphoma kinase rearrangements in lung cancer with nodular ground-glass opacity. BMC Cancer.

[21] Hiramatsu M, Inagaki T, Inagaki T, Matsui Y, Satoh Y, Okumura S, Ishikawa Y, Miyaoka E, Nakagawa K (2008). Pulmonary ground-glass opacity (GGO) lesions-large size and a history of lung cancer are risk factors for growth. J Thorac Oncol.

[22] Lee SW, Leem CS, Kim TJ, Lee KW, Chung JH, Jheon S, Lee JH, Lee CT (2013). The long-term course of ground-glass opacities detected on thin-section computed tomography. Respir Med.

[23] Heo EY, Lee KW, Jheon S, Lee JH, Lee CT, Yoon HI (2011). Surgical resection of highly suspicious pulmonary nodules without a tissue diagnosis. Jpn J Clin Oncol.

[24] Hur J, Lee HJ, Nam JE, Kim YJ, Kim TH, Choe KO, Choi BW (2009). Diagnostic accuracy of CT fluoroscopy-guided needle aspiration biopsy of ground-glass opacity pulmonary lesions. AJR Am J Roentgenol.

[25] Infante M, Lutman RF, Imparato S, Di Rocco M, Ceresoli GL, Torri V, Morenghi E, Minuti F, Cavuto S, Bottoni E, Inzirillo F, Cariboni U, Errico V, Incarbone MA, Ferraroli G, Brambilla G, Alloisio M, Ravasi G (2009). Differential diagnosis and management of focal ground-glass opacities. Eur Respir J.

[26] Whitson BA, Groth SS, Duval SJ, Swanson SJ, Maddaus MA (2008). Surgery for early-stage non-small cell lung cancer: a systematic review of the video-assisted thoracoscopic surgery versus thoracotomy approaches to lobectomy. Ann Thorac Surg.

[27] Watanabe S, Watanabe T, Arai K, Kasai T, Haratake J, Urayama H (2002). Results of wedge resection for focal bronchioloalveolar carcinoma showing pure ground-glass attenuation on computed tomography. Ann Thorac Surg.

[28] Godoy MC, Truong MT, Sabloff B, Naidich DP (2013). Subsolid pulmonary nodule management and lung adenocarcinoma classification: state of the art and future trends. Semin Roentgenol.

[29] Farooqi AO, Cham M, Zhang L, Beasley MB, Austin JH, Miller A, Zulueta JJ, Roberts H, Enser C, Kao SJ, Thorsen MK, Smith JP, Libby DM, Yip R, Yankelevitz DF, Henschke CI (2012). Lung cancer associated with cystic airspaces. AJR Am J Roentgenol.

[30] Kim HY, Shim YM, Lee KS, Han J, Yi CA, Kim YK (2007). Persistent pulmonary nodular ground-glass opacity at thin-section CT: histopathologic comparisons. Radiology.

[31] Nakata M, Saeki H, Takata I, Segawa Y, Mogami H, Mandai K, Eguchi K (2002). Focal ground-glass opacity detected by low-dose helical CT. Chest.

[CR32] The pre-publication history for this paper can be accessed here:http://www.biomedcentral.com/1471-2407/14/838/prepub

